# A dynamic model for tumour growth and metastasis formation

**DOI:** 10.1186/2043-9113-2-11

**Published:** 2012-07-05

**Authors:** Volker Haustein, Udo Schumacher

**Affiliations:** 1 Institute of Anatomy and Experimental Morphology, University Hospital Hamburg-Eppendorf, Martinistraße 52, D-20246, Hamburg, Germany

**Keywords:** Breast cancer, Computational calculations, Gompertzian growth function, Tumour growth models, Metastasis formation

## Abstract

A simple and fast computational model to describe the dynamics of tumour growth and metastasis formation is presented. The model is based on the calculation of successive generations of tumour cells and enables one to describe biologically important entities like tumour volume, time point of 1^st^ metastatic growth or number of metastatic colonies at a given time. The model entirely relies on the chronology of these successive events of the metastatic cascade. The simulation calculations were performed for two embedded growth models to describe the Gompertzian like growth behaviour of tumours. The initial training of the models was carried out using an analytical solution for the size distribution of metastases of a hepatocellular carcinoma. We then show the applicability of our models to clinical data from the Munich Cancer Registry. Growth and dissemination characteristics of metastatic cells originating from cells in the primary breast cancer can be modelled thus showing its ability to perform systematic analyses relevant for clinical breast cancer research and treatment. In particular, our calculations show that generally metastases formation has already been initiated before the primary can be detected clinically.

## Background

In the mathematically oriented medical literature different models are applied to describe the process of tumour growth and metastasis formation. Most of these models fall in one of the three following categories: The first ones are discrete models on the basis of single cell interactions which are then described by the aid of M^te^ Carlo simulations. The second ones are complex mathematical analyses of continuum models on the base of differential equations. A good overview of these approaches can be found in the articles of Ward and King [1,2] and Roose, Chapman and Maini [3]. A third interesting alternate ansatz was developed by Iwata, Kawasaki and Shigesada [4,5] which is in the following referred to as the IKS-model. They model metastasis formation from the primary tumour and from metastases from metastases and give complex analytical solutions for the density respective the abundance of metastatic colonies depending on different growth functions of the primary tumour.

All the abovementioned methods have the disadvantage of complex re-analysis or the need for time consuming numerical re-calculations when input functions or constraints are to be varied. Systematic investigations and the analysis of metastasis modulating events or treatment effects upon metastasis formation are limited due to the complexity or the computing power required.

In the following a mathematical model is presented which is based upon a series of successive generations of tumour development. This model enables a fast calculation of macroscopic relevant entities of the metastatic cascade. The entire programming was carried out in the C language using the graphical analysis package ***root***, developed at CERN [6].

## Results

### The computational model

Metastasis formation is a complex process often referred to as a cascade as each step has to be performed in a certain order. It is initiated, when the first primary malignant cell starts to proliferate. If the developing primary tumour has reached a certain size, it sends out angiogenetic signals and blood vessels grow into the primary tumour. The future metastatic cell has to dissolve itself from the tumour mass by loosening of cell to cell contacts and has to degrade the basal lamina and the surrounding connective tissue. Having achieved this step in malignant progression, the future metastatic cell has to enter the bloodstream by migrating through the blood vessel endothelium. Once arrived in the circulation, the future metastatic cell has to survive in it and has to attach to the endothelium in the organ of the future metastasis. After attachment to the endothelial cell, the cell has to transmigrate through the endothelium and has to lodge in the stroma of the host organ. Presumably under the influence of local growth factors, the metastatic tumour cell has to proliferate in order to become a clinically detectable metastasis.

The characterized cascade can be effectively modelled by following this chronology of the events and making some realistic assumptions on the underlying distribution functions. This approach will be outlined in the following.

At each stage or generation of development a malignant cell inside a tumour has three possibilities: mitosis with doubling, apoptosis or migration into the next compartment where it becomes a potential metastatic cell. Each of these processes follows an exponential distribution with a characteristic constant λ_a,m,d_ = log(2)/T_a,m,d_. With the restriction of no overlap in time, that implies that the 1^st^ started process will be executed, this results in a common exponential with λ_G_=λ_d_-λ_a_-λ_m_ and a time per generation T_G_ = log(2.d)/λ_G_. The fractions λ_a,m,d_/λ_G,_ takes the values a,d and m and fulfil the constraint a + d + m = 1; the numbers are not necessarily constant over all considered generations. After n cycles this leads to (2.d)^n^ tumour cells. The number of potential metastatic cells is simply ∑(2.d)^(n-1)^·m. Either taking m(n) = m·δ^n^ or for calculation purposes more convenient leaving m constant and multiplying with a power of the actual number of cells, a metastasis formation process proportional to tumour volume V (δ=1), surface V^2/3^ or diameter V^1/3^ (δ<1) can be realized. Different interactions in the environment of the tumour or inside the lymphatic or blood vessel system will then lead to a finite life cycle of these disseminated cells either while being killed by the immune system respective by apoptosis or due to successful **c**olonisation into the stroma of a peripheral organ. Again we assume an exponential distribution; now with the decay constant λ_env_= λ_k_+λ_c_.

In continuation of the generation model with the time steps T_G_ we have to distinguish between cells that just enter the circulation and those that have already populated the blood or lymphatic system. The later group are surviving cells originating from former generations which had already entered circulation prior to the actual time step T_G_. These cells will simply be successively reduced by a factor F = exp[−λ_env_·T_G_]. Accordingly the part (1-F) will be eliminated from the blood system. In our model only the small fraction λ_c_/λ_env_ of these cells will each colonise and develop metastases. The mean time point can be calculated by integrating the distribution function of such an exponential decay. The other group of cells, cells that just enter the circulation are subject to a different treatment. The process of creation and immediate elimination during the same time step T_G_ has to be accounted for. Number of surviving cells as well as mean time point and number of colonizing cells can be calculated by the combination of both the distribution function for dissemination into the blood stream and function for subsequent colonization of the stroma. Especially when the time scales for the life cycle T_env_ in the environment respective the generation time T_G_ differs significantly, this approach is necessary to calculate a more precise time of 1^st^ metastasis formation.

From the computational point of view simply a loop over N generations was generated, where each cycle generates the cell size of the primary tumour, number of disseminated cells in the blood or lymphatic vessels and the number of metastatic cells per T_G_ and in total at time n·T_G_. Following the same strategy and by the use of recursion techniques the development in time of per T_G_ released metastatic cells and the process of secondary respective multiple metastases formation from metastases was calculated. If not indicated otherwise the further calculation is performed under the assumption, that metastases grow with the same speed and to the same maximal tumour size as the primary tumour.

### Modelling tumour growth: the Gompertz-function

In the following we will demonstrate the features of our straightforward strategy using the widely used Gompertzian growth function given by g(x)=μ⋅x⋅log(b/x). The parameter b is the asymptotic maximal reachable cell or tumour size and μ is the growth constant. Integration gives a tumour size of G(t) = b(1-exp(-μ∙t)). For metastasis formation a rate of the following form was taken by IKS: β(x) = γ·x^α^. The parameter γ is simply the colonization coefficient and α stands for the fractal dimension of blood vessels infiltrating the tumour. In principle α denotes the fraction of tumour cells which participate in metastasis formation. For example α = 2/3 reflects a superficial angiogenesis of the tumour and dissemination occurs then notably only from the surface.

**Figure 1:**
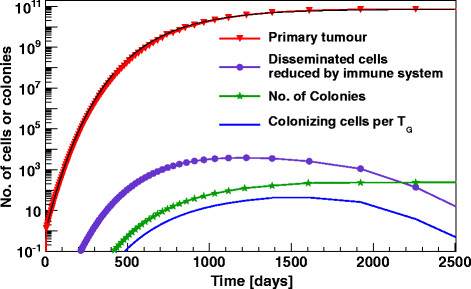
**Dynamic of tumour growth and metastasis formation in the MS-model.** Tumour development and number of disseminated cells at different steps of the simulation as a function of time. The calculations were done for a real hepatocellular carcinoma; maximal reachable tumour size and growth rate was originally fitted by the IKS-model with a Gompertzian growth function. The rates for metastasis formation in the MS-model were adjusted to give the same number of visible colonies as the analytical solution of the IKS-model.

From the above given equation for G(t) the initial characteristic doubling time T_D_ can be calculated to T_D_ = -1/μ∙log(1-log(2)/log(b). In our model only the fraction d leads to further tumour growth, this simply translates to T_G_ = T_D_⋅log(2.d)/log(2) = log(2.d)/λ_G_. To realize the Gompertzian like bending behaviour two different models were used. In the 1^st^ model - the **M**etabolic **S**tagnation model (MS-model) - a per generation variation of T_G_ with log(b)/log(b/x) was taken, where x denotes the tumour size before starting a new generation of tumour cells. In the 2^nd^ model, a continuous decreasing of the doubling rate was assumed; the numbers can be calculated by a fit to a given Gompertzian growth. With the constraint a + d + m = 1 this reduction is compensated by a successive increasing of the number of apoptotic cells. This approach lets the generation time constant over the entire live cycle of the tumour and will be referenced to as the model of **G**eneration **D**ependent **R**ates (GDR-model). Our approach takes the biology of the cell cycle into account and represents an approximation of the Gompertzian growth. Especially the S-shaped bending in the saturation region of the growth curve is not perfectly reproduced. An over-estimate of the tumour volume of up to 10% depending on the weights during the fit procedure remains for the GDR-model. The MS-model fits marginally better, but possesses the same tendency. Figure 1 shows the simulation results for a hepatocellular carcinoma fitted by the IKS-model [4]. In this specific case chemotherapy started 639 days after initial diagnosis of the primary tumour. Due to this long onset, information about nearly undisturbed tumour growth as well as number and growth of metastases, which were detected for the 1^st^ time on CT-images 432 days after primary diagnosis, are available. The values which we adopted from IKS were the cell size b = 7.3⋅10^10^ cells and μ=0.00286 day^-1^, which leads to an initial doubling time T_D_ of 9.8 days. As aforementioned in the 1^st^ simulation step of our model of successive generations of tumour cells three concurrent processes are considered: doubling, apoptosis and dissemination respective migration into the next compartment. The branching ratio for cell doubling d to the combined term of apoptosis and migration a + m was set to 2:1. This somewhat arbitrary choice has the advantage of both, smaller simulation time steps T_G_ because of the relation T_G_ = T_D_⋅log(2.d)/log(2) and the possibility of a variation of the rate for migration in a wide range with respect to the constraint a + d + m = 1. Dissemination from the primary occurs at a calculated ratio of m = 2.49⋅10^-6^ to get the identical number of metastases as given by IKS for day 432 after diagnosis at a colony size of ~4.6⋅10^7^ cells. In the 2^nd^ simulation step the disseminated and potential metastatic cells will be followed up. The ratio for elimination by the immune system respective successful colonisation was taken to 10^-4^ at a mean lifetime of the tumour cells in the bloodstream of 1 day. Tumour growth and the different simulation steps of the developing cascade are shown for the MS-model. The tumour growth function is in return underlayed with a fit of the Gompertzian growth with b = 7.321⋅10^10^ cells and μ=0.00296 day^-1^. The full blue line shows the number of per generation colonizing cells with the characteristic maximum at the time, when the variation of the Gompertz function has its maximum d/dt[dG/dt] = 0. The green asterisk stands for the cumulative numbers of 1^st^ order colonies.

**Figure 2:**
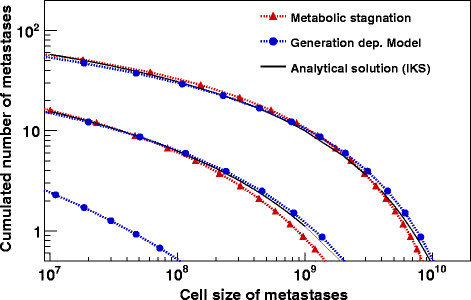
**Comparison of the cumulated number of metastases in the MS- and GDR-model with the IKS-model.** Cumulated number of metastases derived from the primary tumour and the 1^st^ order metastases for the MS- and GDR-model with the analytical solution from IKS-model. The days 432 (lower band) and 632 (upper band) after diagnosis of the primary tumour were chosen, which occurred 678 days after the initiation of the tumour. The clinical data were initially fitted by IKS for a hepatocellular carcinoma following the Gompertzian growth function with a rate for metastasis formation proportional to V^.663^. To get an impression of the influence of the metastases from metastases formation the contribution of the 2^nd^ order metastases is shown separately for the GDR-model in the lower left corner. The 2^nd^ order metastases formation from MS is below the chosen range.

**Figure 3:**
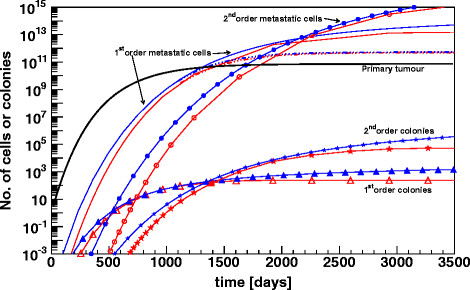
**Metastasis formation: number of colonies and metastatic tumour mass.** Total amount of metastatic cells as well as total number of 1^st^ and 2^nd^ order colonies. The blue filled symbols represent the GDR-model, the open red stand for the MS-model. The growth function of the primary is also shown; an excision immediately after diagnosis would lead to a decrease of the amount of metastatic cells by 2 decades (dashed-dotted lines). Around day 1300 the amount of metastatic cells equals the primary, two years later the hundred fold is reached and latest from now the 2^nd^ order metastases would dominate the course of disease.

The broad accordance of our rather simple model with the analytical solution of IKS is shown in Figure 2, where the cumulated number of metastases from the primary and higher, up to the 3^rd^ order metastases is plotted for both models. In the original article [4] a complex solution for the density of metastases and meta-metastases is given. The cumulated number of colonies of a given size can be obtained by integrating the density and taking the positive real and the adjacent 16 complex residues for the calculations; please refer to the original article for a deeper understanding of the details of the analytical solution. Different to the MS-model, where the same set of parameters were used as shown above, in the GDR-model the 1^st^ of both metastasis steps was assumed to be proportional to V^2/3^. This is in accordance with the IKS-model where the fit gives a value of 0.663 for the fractal dimension α. The initial rate for doubling d_i_ = 2/3 was selected to be identical as the one for the MS-model. The difference between d_i_ and the final d_f_ = 1/2 was then obtained by a step by step decrease of d_i_ by 1.23%. This value was calculated by a fit of our approach to the given Gompertzian function. Due to the normalization procedure at a colony size of ~4.6⋅10^7^ cells the rate for dissemination from the primary tumour was calculated to be m = 1.17⋅10^-3^. The combination of the ratios for dissemination and the 10^-4^ for colonisation in the GDR-model is comparable with the 5.3⋅10^-8^ day^-1^ given by IKS. Both models are in good agreement with the analytical solution on day 432 but also on day 632 when metastases had progressed. Differences can be observed when the total number of clinically not detectable metastases including single cells was examined. Both of our models reach only a level of ~70% as compared to the analytical solution. These under-estimates are due to the above-mentioned systematic differences between the Gompertzian function and our approximations. Nevertheless, both models fit the clinical data of the hepatocellular carcinoma with high precision, which is remarkable as different proportionalities for the calculation of metastases formation were used. From a macroscopic point of view, the MS-model seems to “simulate” metastasis formation proportional to the surface of a tumour. A more detailed view of the dynamics with the same parameter set as above is shown in Figure 3. The total numbers of colonizing cells from the primary and from metastatic tumours are plotted together with the total amount of metastatic cells. The red open symbols stand for the MS- and the filled blue symbols for the GDR-model. About 22 month after initial diagnosis the overall cell size of the 1^st^ order metastases reaches the tumour mass of the primary, about 2 years later this accumulates to the hundredfold primary tumour mass. This calculation corresponds to the time, when the 2^nd^ order metastases would become clinically important. At this time point they provide a tumour mass comparable to the total mass of the 1^st^ order metastases. Of course this calculation only corresponds to the patient if there are no clinical interventions such as surgical removal of the primary tumour and that metastases grow at the same rate as primary tumours do. An excision or a total embolisation of the tumour at the earliest time would lead to a significant decrease of 1^st^ order metastatic tumour mass of about 2 decades (dashed-dotted lines).

In the previous paragraph we proposed two models that describe a mitotic behaviour variable in time, but emanate from biologically totally different approaches. Both models show a Gompertzian like tumour growth and reproduce the metastasis formation of a given hepatocellular carcinoma. Before demonstrating the validity for breast cancer research we next show model systematic spread.

### Systematic investigations

**Figure 4:**
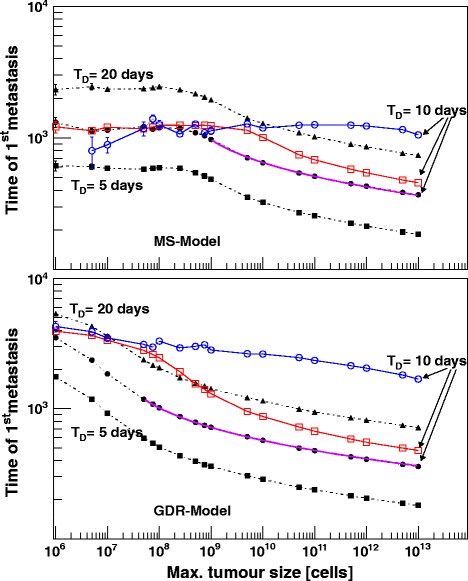
**Time point of 1**^st^**metastasis formation for the MS- and GDR-model.** Time of 1^st^ metastasis formation as a function of maximal tumour size b of the Gompertz function. Results for the MS-model are shown in the upper, those for the GDR-model in the lower part. The simulations were calculated for three different rate parameters μ, which corresponded to an initial doubling time of 5, 10 and 20 days at a reference cell size of 10^11^, respectively. The rates for metastasis formation were chosen the same as in Figures 2, 3 (black symbols) or reduced by one tenths (red symbols). Dissemination occurred proportional to V (MS-model) and V^2/3^ (GDR-model). For the blue graphs a reduced dissemination (V^2/3^ and V^1/3^) was expected.

From the clinical point of view the most pressing question to be answered by this model is: when does the 1^st^ malignant cell spread out to form a distant metastasis? In the two parts of Figure 4 the mean time T^1stM^ is plotted against the maximal tumour cell size denoted by the parameter b. Each data point consists of 2000–10000 entries, based on randomized “one colonizing cell” events, taken from the former generated metastases distribution function. Tumour growth was followed up for about 25 years; at least for primaries with cell size ≅10^9^ metastasis formation will occur in all patients.

As can be deduced from the above given equation for G(t) the characteristic bending of the Gompertzian curve depends both on the maximal tumour size b and the rate parameter μ. On the other hand a constant μ translates in our model into a slightly b dependent initial T_D_. For our calculations we choose a T_D_ of 5, 10 (comparable to the 9.8 days from IKS) and 20 days at a reference cell size of b = 10^11^. To be comparable with the previous results dissemination from the primary occur proportional to V for the MS- respective V^2/3^ for the GDR-model. All rates and the lifetime of the tumour cell were chosen the same as in Figure 2, 3. A lifetime of 1 day is small or comparable to T_D_ and T_G_; hence a realistic chance for colonisation into the stroma is given only for the time step being disseminated or the following one. A variation of the lifetime of a malignant cell within reasonable limits leads therefore simply to a scaling up of the combined rate of dissemination and colonisation. Our calculations confirm this assumption; systematic effects except those that can be seen by a variation of the combined rate were not regarded.

In the upper part of Figure 4 results are shown for the MS-model for the three different developments of the tumour growth with time given by T_D_ = 5, 10 and 20 days (dashed-dotted lines with black filled circles). In the lower part of Figure 4 the corresponding data for the GDR-model are presented. To demonstrate that systematic differences between our models exist we include in both parts two further curves: The red lines with open circles represents a by one tenths reduced tumour growth rate, the blue lines and circles show the dissemination step following the next logical lower power of V^m^, corresponding to the series volume, surface and diameter. For small maximal tumour sizes within the MS-model, T^1stM^ takes a constant and depends only on the bending behaviour of the tumour growth curve with time. The graphs with the reduced rate and the lowered dependency to V^2/3^ underline this strong correlation. The value for T^1stM^ fits reasonable well with the time when the variation of the Gompertz-function has reached its maximum, or when it is expressed with an equation when d/dt[dG/dt] = 0. Since the dissemination step is coupled to mitosis this corresponds to the time-point when maximal metastases formation occurs. The probability for metastases formation is for small maximal cell sizes b in the percentage level and increases to 1 at around b = 10^9^ cells. It is obvious that the MS-model would be able to describe a tumour entity which shows an extreme early but low rate of metastasis formation. In particular, in the next section we will argue that the MS-model is a suitable candidate to describe a subgroup of breast cancer data. The monotonous decreasing data points above 10^9^ cells and the range b ≫ 5⋅10^7^ cells for the GDR-model can be fitted with f(b) = −γ/μ⋅log(1-log(β)/log(b)); a functional relation which is already known in a similar form from the combination of μ with T_D_. The parameter γ is simply a scaling factor and β depends both on μ or T_D_ and the dissemination characteristics V^m^. For the GDR-model the comparison of the three graphs of T_D_ = 10 days but with different rates or dissemination characteristics emphasize again that a common asymptotic value for T^1stM^ will be reached if small tumour sizes are considered. Different to the MS-model, the GDR-model T^1stM^ does not correspond to the maximum of the variation of the Gompertzian growth. If the follow up time is sufficiently long, metastases formation will occur even if the primary does not change its size any longer as it has reached its maximum size. In contrast to the MS-model where metastases start early and with low rate the metastases in the GDR model will colonize relative late but more frequent. This metastatic pattern reflects the fundamental differences between the two models. On one hand we assume a continuous prolongation of the tumour generation time T_G_. This involves a naturally ageing of the cells with slower and slower running processes but with a regular and balanced sequence in mitosis and apoptosis. On the other hand we have highly active tumour cells; T_G_ remains constant but everything run with a high and lethal error rate. The fraction for doubling and apoptosis are shifted against each other which results in the decreasing tumour growth. Both models find their analogy in the biology of cells. It is known that a misbalance of anabolic and metabolic processes, reduced concentrations of enzymes or a failure in signal transduction is jointly responsible for an ageing of cells. Inadequate repair mechanism or missing stop signals in time during G_0_-phase of mitosis on the other hand leads to a slightly increasing of unformed and later apoptotic cells.

### A clinical application in breast cancer

**Figure 5:**
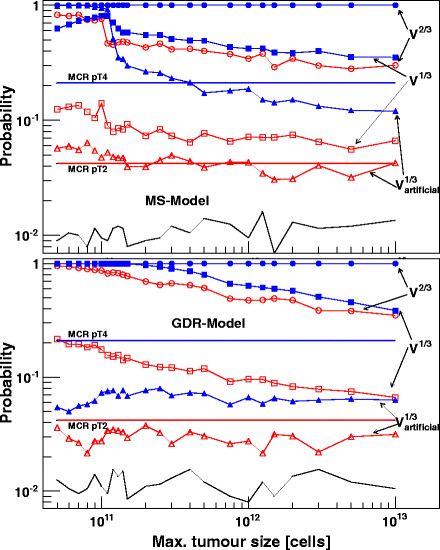
**Probability of metastasis formation for tumours of pT2 and pT4-stage.** Simulation results for the probability of metastasis formation as a function of maximal reachable tumour size b of the Gompertz function. Calculations are shown for dissemination of malignant cells from the primary proportional to V^2/3^ and V^1/3^; the MS-model is shown in the upper, the GDR-model in the lower part. For the V^1/3^ proportionalities models with artificial, but reasonable reduced growth of the metastases are included. The black and red dashed lines denote the Munich Cancer Registry probabilities for pT2 and pT4. The normalisation to 1.1% at pT1-stage (black line) was calculated for the entire interval [5⋅10^10^, 10^13^ cells] of maximal tumour sizes; only one typical curve is shown for each plot. For every data point the mean of 2000 randomized courses of disease, each based on integer-disseminated cells, was calculated.

After having adjusted our mathematical model to the IKS data, we wanted to expand its application to the breast cancer data from the Munich Cancer Registry (MCR) [7,8]. We followed their argument that the mean age of women, who have different pTx category at the time of initial diagnosis of breast cancer, reflects the mean tumour growth. This assumption should at least be valid for small tumours of the pT1 and pT2 categories. Expecting a Gompertzian growth function, the initiation of tumour growth and the bending behaviour for different maximal tumour cell sizes can then be fitted. The data from Munich cancer registry give a mean age of 57 years for pT1 and 58.1 years for pT2 indicating a mean time of 1.1 years for progression from pT1 to pT2. Metastasis formation at time of initial diagnosis was observed in 1.1% of the pT1 cases and 4.2% for pT2 cases. No discrimination was made concerning histological grade, oestrogen-receptor positivity or lymph node involvement. It must also be noted, that the mean age of pT3 patients with 55.9 years is younger then that of both pT1/2 patients and pT4 patients which showed an unexpectedly high mean age of 65.3 years. Without studying the age distributions inside the groups in detail we have no definite explanation why pT3 tumours do manifest at younger-aged patients. One reason could be that an extremely aggressive or fast growing subgroup of cancer is responsible for this effect. The relative high proportion of G3 and oestrogen-receptor-negative cases points toward that explanation. Particularly the small numbers of 671 patients for pT3 (5772 for pT1, 4897 for pT2, 1092 for pT4) are surprising. Because of these inconsistencies within the age distribution data from pT3/4 patients were not taken into consideration. Nevertheless the numbers of cases with metastasis formation with 9.7 and 21%, respectively, should be kept in mind, as they could give some clues about the development in time for an untreated pT1/2 tumour. For pT1 tumours a mean diameter of 14 mm, 28 mm for pT2 and 60 mm for pT4 were given [7].). The volume of a single cell was assumed to be 10^3^ μm^3^. To investigate whether our results depend crucial on this estimate, calculations were done with different quotients of the volume of a primary and a metastatic cell; the results are summarized in Table 1. The diameter of visible metastases was expected to be 4.57 mm corresponding to a colony size of 5⋅10^7^ cells, if primary and metastatic cells are equal in volume, or 1⋅10^8^ cells, if a metastatic cell has only half of the volume of a primary tumour cell. In Figure 5 the probabilities for metastases formation at different stages of tumour development are plotted against the maximal reachable tumour size b. Each data point represents the mean of 2000 randomized courses of disease, each based on integer-disseminated cells taken from the former simulated time distribution of the colonizing metastases. The data are normalized to the abovementioned 1.1% at pT1 stage (black line). A comparison with the rates used for the hepatocellular carcinoma shows a considerable agreement between the two tumour entities. For example, a breast cancer primary with 7⋅10^10^ cells in the asymptotic region and dissemination proportional to V^2/3^ as used in Figures 1,2,3 shows only a 2.5 higher factor for the rates of 3.6⋅10^-7^ day^-1^ for the GDR-model than those used for the hepatocellular carcinoma in the IKS model. The red markers and lines in Figure 5 show the calculated probabilities for metastasis formation at time of 1^st^ diagnosis for pT2 category. The blue lines and symbols represent the calculated probabilities for tumours of 60 mm in diameter, which only corresponds to the mean size of a pT4 tumour and not to the mean age information as given by the Munich Cancer Registry. Nevertheless, for reasons of simplicity in the further context we will speak of pT4-stage tumours.

**Table 1:** **Probability and time of 1**^**st**^**metastasis formation**

				MS-Model				GDR-Model			
Dissemination from primary	Metastases growth characteristis	Single Cell size [10^3^ µm^3^]		Metastasis Formation [%]			Mean time of 1^st^ colonizing cell [months]	Metastasis Formation [%]			Mean time of 1^st^ colonizing cell [months]
		Prim.	Meta.	Visible at pT2	Visible at pT4	Before pT1		Visible at pT2	Visible at pT4	Before pT1	
V	P	1	1	95.0	100.0	100.0	22.6	99.5	100.0	100.0	23.7
		2	1	93.3	100.0	100.0	22.1	97.0	100.0	100.0	23.3
V^2/3^	P	1	1	36.6	100.0	100.0	27.5	48.2	100.0	100.0	28.3
		2	1	32.3	100.0	100.0	27.5	40.5	100.0	100.0	28.1
		1	0.5	41.2	100.0	100.0	23.9	60.7	100.0	100.0	24.7
	A	1	1	10.6	87.9	100.0	16.2	5.5	22.7	71.6	13.4
		2	1	9.5	79.6	100.0	17.1	5.8	24.2	89.1	14.1
		1	0.5	10.3	90.5	100.0	14.5	6.0	25.2	77.7	11.9
V^1/3^	P	1	1	6.9	42.4	45.9	53.1	9.4	63.9	61.2	45.2
		2	1	6.6	44.4	59.2	51.2	8.4	58.2	76.6	45.2
		1	0.5	7.9	54.1	63.9	49.4	10.9	75.4	80.3	40.0
	A	1	1	4.0	17.3	98.1	29.5	2.5	6.1	15.2	22.7
		2	1	4.5	17.2	99.3	30.7	3.5	7.9	22.1	23.6
		1	0.5	5.0	20.2	99.8	25.8	3.5	8.7	19.9	19.6

In the upper part of Figure 5 simulation results are shown for the MS-model with dissemination proportional to V^2/3^ and V^1/3^, the lower part shows the results for the GDR-model, again with a V^2/3^ and V^1/3^ dependency. For both models a variation of the growth characteristics with an artificially slewed down growth behaviour of the metastatic cells are calculated and included in the plots. For the GDR-model this growth behaviour was achieved by starting the metastatic growth process with the doubling rate of the time step the malignant cell has being disseminated. Taking the same generation time T_G_ and error rate for mitosis as for the primary tumour, a reduced initial doubling rate consequently leads to a reduced maximal colony size of the metastases. The modified growth function for the MS-model was achieved by starting the metastatic growth just with the prolonged T_G_ of the generation the cell was disseminated. The ageing process is inherited. These colonies will reach the same maximal size as the primary but need for this growth significantly more time. Figure 5 clearly demonstrates that the metastases formation rate of 4.2% at time of initial diagnosis for pT2 patients and also the 21% for the pT4 patients can hardly be reached. Dependencies as V^1/3^ for the MS-model or even lower ones for the GDR-model are needed for the dissemination step, in order to achieve partly congruence between the clinical breast cancer data and our calculated probabilities.

Tumours of pT1 stage are in diameter just 3 times larger than our current clinical detection limit. To detect metastases of nearly equal size as the primary tumour indicates that the metastasis initiating cell must have been disseminated extremely early during tumour progression and even more importantly at a considerable rate of spread. Consequently the numbers of metastases will then increase nearly exponentially. Some mouse models [9] suggest an extremely early start of the dissemination process and are at least therefore in good agreement with our calculations. For reasons of clearness we recapitulate our assumptions: the whole data sample can be described by a single model and pT1 and pT2 mean age reflects the Gompertzian like tumour growth. Then it is mandatory, that either proportionalities ≤V^1/3^ should be taken into account to describe the low clinical probabilities for metastasis formation or the growth characteristics of metastatic cells should be different from that of the primordial stem cell initiating the primary tumour. Our models with the artificially reduced growth functions of the cells within metastases apparently come into the range of the clinical data. Due to the reduced maximal colony size in the variation of the GDR-model metastases do not become large enough to be clinically detectable and the probability for the presence of metastases at stage pT2 is around 3%. At pT4 the probability falls below 10% where 21% was given for the MCR data. This indicates that the reduced growth as chosen for the GDR model somewhat underestimate the real growth characteristics. Anyhow it clearly demonstrates that a similar mechanism would be helpful to reproduce the data. More favourable is the situation for the MS-model. Metastases that colonize distant organs at a later stage of malignant progression grow much slower than the primary tumour initially did. They remain hidden for a long time (“dormancy”). Under these assumptions we achieve a fair accordance with the data from the cancer registry, both pT2 and pT4-stage probabilities are reproduced. The complete data set is summarized in Table 1, given are the mean values for the interval [7.5⋅10^11^, 1.25⋅10^12^ cells] of the maximal reachable tumour size.

We have verified that a variation of the single cell size of both, primary and metastatic cells in the same direction do not lead to any noteworthy shift in the probabilities of metastasis formation. This is mainly due to the normalization at pT1-stage. To show that our method is in general insensitive to the exact size of a tumour cell calculations where the quotient of metastatic and primary cell diameter varies by a factor ±2 are also given. Small systematic effects can only be seen for the standard growth behaviour of the metastatic cells. A reduction of the metastatic single cell size to half volume results in a relative 5-10% higher probability for primary metastasis formation at the pT2-stage. This is a direct consequence of the reduced visibility during the normalisation procedure to 1.1% at pT1-stage. On the other hand, doubling of the primary single cell size leads to a smoother bending of the tumour growth curve with a reduced growth rate. The increase in metastasis formation between pT1 and pT2 will therefore also be reduced. Again a 5-10% effect relative to the standard values can be seen.

In the following we will focus on those models in which metastases grow like the primary tumour. The time a primary tumour of the considered size (10^12^ cells) needs to reach the pT1 stage is single cell size dependent 49.4 month for cells of 2**⋅**10^3^ μm^3^ in volume, respective 54.9 month for 10^3^ μm^3^ cells. The mean time for the 1^st^ malignant cell to colonize into the stroma of the target organ lay around 23 month for the V-dependency and 28 and 45–53 months for the surface and diameter dependencies, respectively. All time distributions have a full width at half maximum of around 70%. These findings indicate that at least in half of the patients metastasis formation has taken place before the primary tumour became visible. The relative survival after 15 years with an over all metastasis formation rate was calculated to 77.6% for pT1 and 24.1% for pT4 [7], not differentiated by any treatment modalities. The MS- as well as the GDR-model with a V^1/3^ dependency for dissemination would best have the ability to explain the data if a reduction of metastases due to radio- or chemotherapy is incorporated.

## Discussion

We proposed a simple model of metastasis formation based on successive series of generations of tumour cells. With relative low computational power our model enables a fast insight into the growth and spreading behaviour of malignant tumours. The modelling itself is independent from the specific growth characteristics of a particular tumour. Here we concentrate on the Gompertzian growth and developed models rooted in the biological behaviour of malignant cells to describe such a growth function. Inside our framework we have demonstrated which mandatory implications can be deduced from the occurrence of metastases at a definite time. Especially the calculations based upon clinical data support the hypothesis that formation of metastases is a continuous and extreme early event during malignant progression. Our results are in good accordance with the analytical solution of Iwata et al. [4], who calculated their model according to a real clinical case. This accordance is remarkable, because we use a simple, straightforward simulation of successive generations of tumour cells whereas the IKS-model is a complex solution for the development in time of the size distribution of metastases. Moreover we have demonstrated that our models should in principle be able to describe the breast cancer data of the Munich Cancer Registry as well. A combination of different V-dependencies for metastasis formation, a small but fast component that dominates the probability at pT1 stage and a slow V^1/3^ dependency for the further observed low numbers at pT2 and pT4 stages should be able to cover the whole range of growth and metastasis pattern. Additional and more detailed clinical data are however necessary before definite statements can be made.

## Conclusions

A novel approach to simulate tumour growth and metastasis formation is presented. Within the framework of our model growth and dissemination characteristics of metastatic cells originating from cells in the primary tumour can be modelled. We adopted our model to clinical breast cancer data thus showing its ability to perform systematic analyses relevant for clinical breast cancer research and treatment. In particular, our calculations using these clinical data show that generally metastases formation has already been happened before the primary tumour can be detected with current clinical methods.

## Abbreviations

IKS = The authors Iwata, Kawasaki and Shigesada; MS = Metabolic stagnation; GDR = Generation dependent rates; MCR = Munich Cancer Registry; pT1,pT2,pT3,pT4 = histopathological TNM classification of malignant tumours.

## Competing interests

The authors declare that they have no competing interests.

## Authors’ contributions

VH did the writing of the programme for the calculations and wrote the first version of the manuscript. VH and US contributed and participated equally in the design of the general outline of the work. US helped in finalising and editing of the final version of the manuscript. Both authors read and approved the final manuscript.
